# Recognition of EEG Signal Motor Imagery Intention Based on Deep Multi-View Feature Learning

**DOI:** 10.3390/s20123496

**Published:** 2020-06-20

**Authors:** Jiacan Xu, Hao Zheng, Jianhui Wang, Donglin Li, Xiaoke Fang

**Affiliations:** 1 School of Information Science and Engineering, Northeastern University, Shenyang 110819, China; wangjianhui@mail.neu.edu.cn (J.W.); 1910309@stu.neu.edu.cn (D.L.); fangxiaoke@mail.neu.edu.cn (X.F.); 2 School of Information Science and Engineering, Shenyang University of Technology, Shenyang 110870, China; zhenghao@sut.edu.cn

**Keywords:** brain-computer interface (BCI), electroencephalography (EEG), multi-view learning, deep neural network, parametric t-distributed stochastic neighbor embedding (p.t-SNE)

## Abstract

Recognition of motor imagery intention is one of the hot current research focuses of brain-computer interface (BCI) studies. It can help patients with physical dyskinesia to convey their movement intentions. In recent years, breakthroughs have been made in the research on recognition of motor imagery task using deep learning, but if the important features related to motor imagery are ignored, it may lead to a decline in the recognition performance of the algorithm. This paper proposes a new deep multi-view feature learning method for the classification task of motor imagery electroencephalogram (EEG) signals. In order to obtain more representative motor imagery features in EEG signals, we introduced a multi-view feature representation based on the characteristics of EEG signals and the differences between different features. Different feature extraction methods were used to respectively extract the time domain, frequency domain, time-frequency domain and spatial features of EEG signals, so as to made them cooperate and complement. Then, the deep restricted Boltzmann machine (RBM) network improved by t-distributed stochastic neighbor embedding(t-SNE) was adopted to learn the multi-view features of EEG signals, so that the algorithm removed the feature redundancy while took into account the global characteristics in the multi-view feature sequence, reduced the dimension of the multi-visual features and enhanced the recognizability of the features. Finally, support vector machine (SVM) was chosen to classify deep multi-view features. Applying our proposed method to the BCI competition IV 2a dataset we obtained excellent classification results. The results show that the deep multi-view feature learning method further improved the classification accuracy of motor imagery tasks.

## 1. Introduction

EEG signals are spontaneous potential activities generated by brain nerve activity and contain abundant brain activity information [1]. The current research on the brain is mainly based on the analysis of EEG signals. The BCI is the key for the human brain to communicate with the outside world. It is a non-muscle channel communication method [2,3,4]. Currently, there are two brain-computer interface technologies, invasive and non-invasive. Non-invasive BCI is widely used because of its convenient operation and low cost [5,6]. Through the non-invasive BCI, we can obtain various patterns of brain activity signals, which are extensively studied and used in signal processing, pattern recognition, cognitive science, medicine, rehabilitation and other fields [7,8]. The analysis of EEG signals can sometimes help patients who cannot act autonomously due to injury to the muscles or nerves that control limb movement, such as strokes, spine injuries, craniocerebral nerve injuries, etc. [9,10]. These patients are unable to control the body autonomously, and in severe cases, even cannot communicate with people. Through EEG signals, patients’ brain activity can be analyzed, helping patients communicate with the outside world, improving their quality of daily life, and reducing their mental burden [11]. Motor imagery can activate the sensorimotor cortex, produce sensorimotor concussion, send out EEG signals, and reflect the subject’s motor intention [2,12]. When the subject imagines a certain part of the limb movement rather than the actual movement, the corresponding reflex area in the human brain will display electrical potential changes. By analyzing the changes in the electrical potential of the EEG signals and recognizing the movement pattern imagined by the current subject, external devices can be controlled to assist the subject to perform the corresponding movement tasks. Therefore, the analysis of EEG signals and the identification of movement intentions are great significance in the field of artificial intelligence rehabilitation medicine.

Regarding how to accurately identify the movement intention of EEG signals, we need to conduct in-depth research on EEG signals. Studies have found that when a subject imagines the movement of a certain limb, the neuron activity will appear in the brain area related to this movement. This energy change is called event-related desynchronization (ERD) and event-related synchronization (ERS) [2,12,13]. The most successful method for detecting ERD/ERS is common spatial patterns (CSP). This is because ERD/ERS is directly related to the sensorimotor rhythm mu and beta, and the frequency band used in the CSP algorithm is mainly 8–30 Hz [14], covering mu and beta rhythma, so CSP is widely and effectively applied in the field of brain-computer interface motor imagery (BCI-MI) [4,15]. Considering the difference in the frequency bands of EEG response of different subjects, the use of fixed frequency bands may result in some useless frequency components and increase the redundant information of features [16]. In response to the frequency-dependent problem of CSP, some frequency band selections of CSP have been proposed, such as FBCSP [17], CSSP [18], CSSSP [19], SBCSP [20] and DFBCSP [21]. These improvements take into account the frequency domain and spatial characteristics of EEG signals, make up for the shortcomings of the CSP algorithm to a certain extent, and show good progress in motor imagery task recognition. However, the information of the motor imagery of EEG signals is reflected not only in the frequency and spatial characteristics, but also in the features of the time domain and time-frequency domain [22]. Therefore, only the frequency domain and the spatial domain are considered, and other domain characteristics are ignored, that may result in the loss of information related to motor imagery, and the lost information may be very helpful for the recognition of motor imagination tasks [2]. In recent years, deep learning has become a research hotspot in various fields, and the use of deep learning to decode EEG signals has attracted the attention of many scholars. Li et al. proposed the hybrid deep structure restricted Boltzmann machine (RBM) [23] and the spatial temporal discriminative restricted Boltzmann machine (ST-DRBM) [24], both of which were applied to the detection of event-related potentials. Lu et al. applied the deep restricted Boltzmann machine to the motor imagery recognition of EEG signals [25], the frequency domain features of EEG signals after FFT or wavelet packet decomposition (WPD) were performed to train the network and then identified motor imaginary task. In the deep RBM network structure, it is usually assumed that the dataset of the top-level network follows Gaussian distribution. However, in actual datasets, abnormal outliers cannot be avoided. Therefore, some problems may occur because the algorithm is sensitive to abnormal data [26].

**Figure 1:**
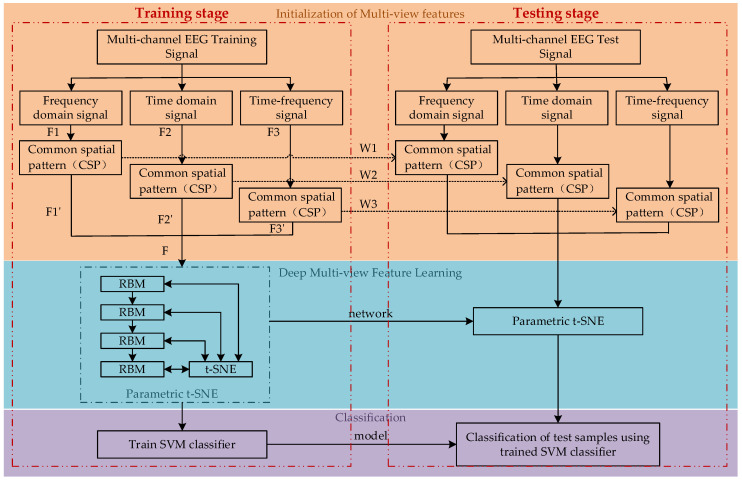
Framework of the proposed method.

In response to the above problems, this paper proposed a new deep multi-view feature learning method. We introduced the multi-view learning framework into the EEG motor imagery task recognition system. The method uses the RBM deep feedforward neural network improved by t-SNE and the CSP to learn the multi-view features of the EEG signal, and utilizes a support vector machine (SVM) to classify and recognize deep multi-view features, the framework of the proposed method is shown in Figure 1. In terms of more completely retaining the EEG information related to the motor imagery, for the time-domain features of the original EEG signals and the frequency-domain and time-frequency features after band pass filtering and wavelet packet decomposition, we adopted CSP to extract their spatial characteristics to establish multi-view features, so that the multi-view features had the time domain, frequency domain, time-frequency domain and spatial characteristics of EEG signals at the same time. We call this part the initialization of multi-view features, as shown in the yellow area in Figure 1. Second, we input the obtained multi-view features into the deep RBM network for pre-training, which reduces the dimensionality of the multi-view features, removing unnecessary information. Then t-SNE was used to correct the trained deep neural network, which solves the crowding problem in latent space, and enhances the inclusiveness of network. This part is shown in Figure 1 as the blue area. We call this deep multi-view feature learning. Finally, we chose SVM to recognize the deep multi-view features of motor imagery, shown in the purple area in Figure 1. This algorithm achieves excellent classification results in the recognition of the four-class motor imagery task for the BCI Competition IV 2a dataset.

This paper is divided into the following sections: The second section describes the algorithm theory and the specific experimental process. The third section shows the results of the experiments. The fourth section discusses these experimental results. The fifth section summarizes this paper.

## 2. Methods

In this section, we introduce the constitution and acquisition method of EEG data used in this paper. Next, the composition of multi-view features was described. Then, the selection basis and experimental process of deep multi-view feature learning algorithm are explained in detail. Finally, the selection strategy of some important network parameters and the classifier were proposed.

### 2.1. Dataset

**Figure 2:**
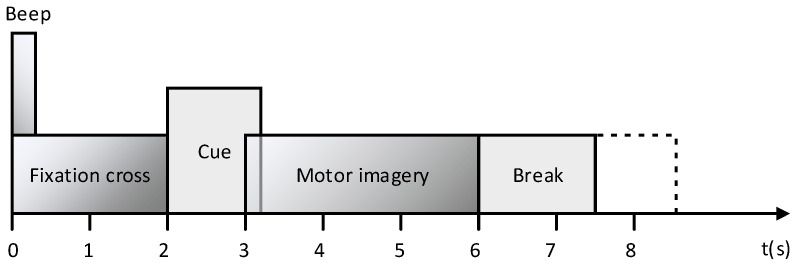
Timing scheme of a trial in BCI Competition IV 2a dataset.

In the experiments, the multi-class motor imagery EEG data came from the BCI competition IV 2a dataset. The dataset was obtained from nine different subjects performing four class motor imaging tasks, including left hand (class 1), right hand (class 2), feet (class 3), and tongue (class 4). The EEG data for each subject consisted of 288 trials of the four-class motor imagery task, and each class of task was tested 72 times. The EEG signals were recorded with 22 channels, sampled at 250 Hz, and band-pass filtered between 0.5 and 100 Hz. The paradigm that single motion imagination task is illustrated in Figure 2.

### 2.2. Initialization of Multi-View Features

In many studies about intention recognition for EEG motor imagery, generally, the original EEG data is directly used as the model training data, but due to the extremely complex EEG signals, which are characterized by unsteady, time-varying, nonlinear and low signal-to-noise ratio, it is difficult to obtain good results by only using the original EEG data for analysis. Therefore, a more appropriate method to analyze EEG signals is by using the feature extraction method. Although traditional feature extraction methods can extract the characteristics of EEG signals, they are usually single. This study used different feature extraction methods to extract the three types of features in the original EEG signal, and extracted their spatial characteristics to form the multi-view features of the EEG signals, the structure is shown in the yellow area in Figure 1. These characteristics of different scales can complement each other to obtain more comprehensive EEG features related to motor imagery.

#### 2.2.1. Time Domain Features

The original EEG signal is the time varying electrical signal of the activity in the group of brain cells collected by the electronic instrument. In the process of acquisition, it was equipped with some noises and artifacts. We utilized EEGLAB toolbox in MatLab to carry out denoising and artifact removal processing on the original EEG signal, and took the processed EEG signal as the time-domain feature, denoted it as $F_{1}$.

#### 2.2.2. Frequency Domain Features

In the analysis of EEG motor imagery, we usually use the 8–30 Hz frequency band, which covers mu and beta sensorimotor rhythms. However, in this single frequency band, some useless frequency components will reduce the classification effect of the system. In addition, the response frequency may vary depending on the different subject. Therefore, this paper used band-pass filters to divide the useful frequency band (8–30 Hz) in the EEG signal into multiple sub-bands, including 8–12 Hz, 12–16 Hz, 16–20 Hz, 20–24 Hz and 24–30 Hz, made it to be the frequency-domain feature $F_{2}$.

#### 2.2.3. Time-Frequency Domain Features

The time-frequency domain describes the frequency characteristics of the signal in different time periods. It is compatible with the time-domain and frequency-domain information of the signal, and can more fully reflect the characteristics of the signal. Wavelet transform is a common method for transforming time-domain signals into time-frequency domain, but it only redecomposes the low-frequency part of the signal when decomposing, and does not continue to decompose the high-frequency part, which makes its decomposition effect on the signal decrease as the signal frequency increases [27]. Fortunately, wavelet packet decomposition can solve this problem exactly. Compared with wavelet decomposition, wavelet packet decomposition has a more detailed division of the time-frequency plane of the signal, and it also has a good analysis effect on the high-frequency parts representing signal details [28]. Therefore, WPD was used to obtain time-frequency features of the EEG signals in this paper.

The common wavelet basis functions include Haar, Daubechies (DBN), Morlet, Meyer, Symlets and so on. Because of the discrete characteristics of EEG signals, Daubechies wavelet was chosen in this paper because it was a tightly supported orthonormal wavelet function [22]. For the order *N* of the wavelet basis function, the larger N value is, the better the regularity of the function is, and the better the result of band segmentation is. However, a higher value of *N* will increase the time of wavelet packet decomposition and reconstruction, which is deteriorates real-time performance. Therefore, the number of wavelet packet decomposition layers was set to 4. The feature decomposed by wavelet packet was used as the time-frequency feature $F_{3}$.

#### 2.2.4. Spatial Domain Features

The signals processed above basically cover the characteristics of different motor imagery. Here we need a way to maximize the difference between different types of EEG signals. CSP is a feature extraction algorithm widely apply to motor imagery of EEG signals [29,30]. This algorithm was commonly employed for feature extraction task of two types of EEG data. Its basic principle was to use diagonalization of matrix to construct an optimal set of spatial filters $W_{csp}$ for projection, and to extract the spatial domain features of EEG signals by CSP, so as to maximize the variance difference between the two kinds of signals [30,31]. And then, the first N rows and the last N rows of the matrix $W_{csp}$ were formed into a matrix W for extracting spatial features and obtain the feature vectors with high discrimination [32].

In the CSP approach, we assume that matrices $X_{1}$ and $X_{2}$ are multi-channel EEG signals under the motor imagery task of two classification. In Equation (1), we can obtain the signal $Z_{i}$ filtered by spatial filter W and spatial characteristics $f_{i}$:

(1) $$\{\begin{array}{c} Z_{i}=W\times X_{i}\\ f_{i}=\log\left(\overset{n}{\underset{t=1}{\sum }}Z_{i}{\left(t\right)}^{2}\right) \end{array},\;\left(i=1,2\right)$$

For four-class motor imagery data, we chose one-versus-rest and two-versus-two strategies. We extracted the spatial characteristics of $F_{1}$, $F_{2}$, and $F_{3}$, respectively, and recorded the extracted features as ${F_{1}}^{\prime }$, ${F_{2}}^{\prime }$, and ${F_{3}}^{\prime }$.

The features ${F_{1}}^{\prime }$, ${F_{2}}^{\prime }$, and ${F_{3}}^{\prime }$ obtained by different feature extraction methods were initialized and matched, and input into the deep learning network as the multi-view feature F, which is defined as:

(2) $$F=\left[\frac{{F_{1}}^{\prime }}{\left||{F_{1}}^{\prime }|\right|},\;\frac{{F_{2}}^{\prime }}{\left||{F_{2}}^{\prime }|\right|},\frac{{F_{3}}^{\prime }}{\left||{F_{3}}^{\prime }|\right|}\right]$$

where ||⋅|| represents the 2-norm.

### 2.3. Deep Multi-View Feature Learning

The multi-view features can more fully reflect the characteristics of the motor imagery of EEG signals, but the spliced multi-view features would increase the dimension of features and augment the computation amount. Therefore, this paper used the parametric t-SNE method to extract the deep features from the multi-view features of the EEG signals, while reducing the feature dimensions and maximizing the difference of the four types of motor imagery. This process is shown in the blue area in Figure 1.

Parametric t-distributed stochastic neighbor embedding (p.t-SNE) is a new parameter dimensionality reduction technique. It is a deep feedforward neural network composed of multi-layer RBMs and backpropagation [26]. It utilized RBM network to learn parameter mapping from high dimensional space X to potential space Y, and adopted t-SNE method to optimize the network in the training [33]. The training process of p.t-SNE dimension reduction algorithm mainly includes the following two stages. First of all, we trained a stack of RBMs, then used the stack of RBMs to construct a pretrained deep neural network, called pretraining. Secondly, the pretrained network was finetuned by using backpropagation which is minimize the cost function that can retain the local structure of the data in the latent space, called finetuning.

#### 2.3.1. Pretraining

In traditional neural networks, the setting of initial weights is very important. When the initial weight was set to larger, the worse local minimum value would be found. When we chose smaller initial weights, the gradient value in the initial layer became smaller, which led to the situation that the multilayer neural network cannot be trained [25,34]. To avoid this problem, we used the deep RBM as the pretraining algorithm for deep learning, which consists of multi-layer RBM network and learns only one layer of features at a time [34].

**Figure 3:**
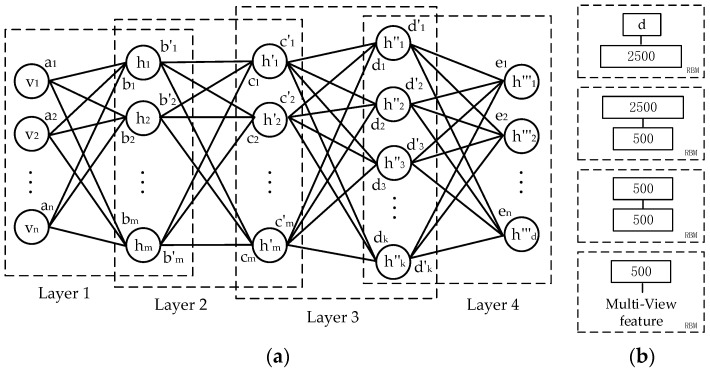
A network structure of Restricted Boltzmann Machine (RBM). (**a**) Schematic diagram of four-layers RBM network; (**b**) Schematic diagram of the number of hidden layer nodes in each layer of RBM network.

In the pretraining, the main work was to train the RBMs stack and then build the feed-forward neural network for pretraining. RBM is an energy-based model with visual and hidden layers [34,35]. The original data is modeled in accordance with the visual node v, and the potential structure of the data is modeled according to the hidden node h. The network structure is demonstrated in Figure 3a. The energy of joint distribution of visible layer and hidden layer is obtained from Equation (3). Where $v_{i}$ and $b_{i}$ represents the visual nodes and its bias, $h_{j}$ and $c_{j}$ represents the hidden nodes and its bias, and $W_{ij}$ represents the weight of the connection between nodes $v_{i}$ and $h_{j}$:

(3) $$E\left(v,h\right)=-\underset{i,j}{\sum }W_{ij}v_{i}h_{j}-\underset{i}{\sum }b_{i}v_{i}-\underset{j}{\sum }c_{j}h_{j}$$

In terms of the special structure of RBM, when the condition of visual nodes is given, the activation condition of each hidden node is conditionally independent, so the activation probability of hidden node j is shown in Equation (4). Since the structure of RBM is symmetric, the activation condition of each visual node is also conditionally independent when the condition of the hidden nodes is given. That is, the activation probability of the visual node i is shown in Equation (5)

(4) $$P\left(h_{j}=1|v\right)=\frac{1}{1+\exp\left(-\sum_{i}W_{ij}v_{i}-c_{j}\right)}=sigmoid\left(c_{j}+\underset{i}{\sum }W_{ij}v_{i}\right)$$

(5) $$P\left(v_{j}=1|h\right)=\frac{1}{1+\exp\left(-\sum_{j}W_{ij}h_{j}-b_{i}\right)}=sigmoid\left(b_{i}+\underset{j}{\sum }W_{ij}h_{j}\right)$$

The parameter model of RBM is learned by making the marginal distribution on the visual nodes under the model, $P_{model}\left(v\right)$, is infinitely approximate to the high dimensional data distribution $P_{data}\left(v\right)$. Since the condition of the visual node under the model is unknown, the RBM model is learned using the contrastive divergence [35]. The contrastive divergence is a fast learning algorithm for RBM that measures the difference between the model distribution and the data distribution through $KL(P_{data}||P_{model})-KL(P_{1}||P_{model})$, where $P_{1}\left(v\right)$ represents the data distribution of the visual nodes that performs an RBM iteration.

In the pretraining, a four layers RBM is applied to layer-wise training which structure is X-500-500-2500-d, as shown in Figure 3b. Where X represents the dimension of the multi-view features F, and d represents the feature dimension after RBM learning. In the training of the RBMs, the sigmoid function was used as the activation function in the first three hidden layers of the network, which makes the hidden layer have Bernoulli distribution. In this part, the learning rate was 0.007, the weight was 0.005, the number of iterations was 70 and the momentum was 0.5 for the first ten iteration and 0.7 for the rest. In the final stage of RBM training, the fourth hidden layer, aiming at making the output of the network more stable, we used the linear activation function. In this section, the learning rate was 0.0002, the weight was 0.003, the number of iterations was 80 and the momentum was 0.6 for the first ten iteration and 0.8 for the rest. Firstly, the multi-view feature F is trained as the input of first layer of RBM network. Then, the contrastive divergence algorithm is used to predict the best value of each hidden layer node. Finally, the value of the hidden node obtained in the previous step is trained as the input data for the second layer of RBM. By this time, the first layer of RBM network training is completed, and this process is iterated to complete the training of the whole multilayer RBM network to provide preparation for the later finetuning stage.

#### 2.3.2. Fine-Tuning

**Figure 4:**
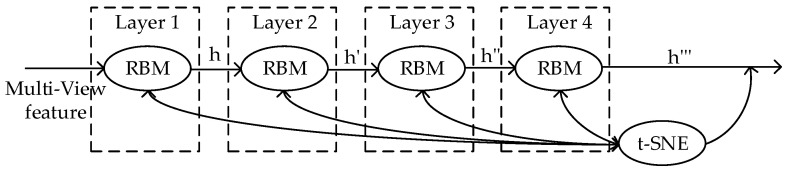
Schematic diagram of t-SNE adjusted the RBM pre-training network.

To obtain more accurate network parameters and more recognizable deep learning features, we adopted t-SNE to adjust the parameters of the pretraining multi-layer RBM network, and the process of backpropagation is presented in Figure 4. In the SNE algorithm, we assumed that the datapoints in the high-dimensional space and the low-dimensional space followed a Gaussian distribution, and used the probabilities between two data points in both the data space and the latent space instead of distances and minimized the difference between them [36]. However, it will reduce the distance of the data points after mapping into the low-dimensional space, which is more serious for two points that are farther away. As a result, after being mapped to the low-dimensional space, the points from different natural clusters in the high-dimensional space are not obvious separated or even aggregated, which is the crowding problem of SNE algorithm [37].

Regarding the solution of the crowding problem, the distribution form of high-dimensional space unchanged, and the Gaussian distribution in low-dimensional space was replaced by the t-distribution [37]. This is because the Gaussian fitting results tend to deviate from the location of most samples when dealing with outliers. The t-distribution is a typical long-tail distribution, and it can retain the contributions made by a small number of outliers. After mapped the data space to the latent space modeled by the feedforward neural network, the term of $q_{ij}$ is defined as follows, where α represents the degrees of freedom of the Student-t distribution:

(6) $$q_{ij}=\frac{{\left(1+||f\left(x_{i}|W\right)-f{\left(x_{j}|W)|\right|}^{2}/\alpha \right)}^{-\frac{\alpha +1}{2}}}{\sum_{k\neq l}{\left(1+||f\left(x_{k}|W\right)-f{\left(x_{l}|W)|\right|}^{2}/\alpha \right)}^{-\frac{\alpha +1}{2}}}$$

Thus, the gradient of the cost function constituted by joint probability with respect to the weight W is expressed as:

(7) $$\frac{\delta C}{\delta W}=\frac{\delta C}{\delta f(x_{i}|W)}\frac{\delta f(x_{i}|W)}{\delta W}$$

Among them:

(8) $$\frac{\delta C}{\delta f(x_{i}|W)}=\frac{2\alpha +2}{\alpha }\underset{j}{\sum }\left(p_{ij}-q_{ij}\right)(f\left(x_{i}|W\right)-f(x_{j}|W)){\left(1+||f\left(x_{i}|W\right)-f{\left(x_{j}|W)|\right|}^{2}/\alpha \right)}^{-\frac{\alpha +1}{2}}$$

To promote the calculation of the joint probability distribution matrix P in the high-dimensional space required in the parameter t-SNE, we need to subdivide the training data into batches. Here, 100 datapoints were selected as one batch, and conjugate gradient algorithm was performed to minimize the difference of joint probability distribution between high-dimensional visual space and low-dimensional potential space. Then 50 backpropagation iterations were adopted to correct the parameters of parameter t-SNE network. For the backpropagation experiments, the perplexity of the conditional distributions $P_{i}$ was set to 25, which is the variance $\sigma_{i}$ of the Gaussian distributions. From this, we get the adjusted network parameters and complete the backpropagation of the deep feed-forward neural network. In the adjustment phase of the network parameters, the freedom degree α of the t-distribution is a parameter that has greater influence on the network. We will discuss how to choose the value of α in the following Section 2.4.

### 2.4. Optimization of Network Parameters

In the entire learning process of the parameter t-SNE, there are two parameters, the dimension d and the freedom degree α, which have a greater influence on the learning result. The d represents the dimension of feature after deep learning algorithm with the parameter t-SNE. The larger the value of d, the more the feature information, but the greater the redundancy. The smaller the value of d, the less the feature information, and also more quickly the subsequent processes, but the smaller d cannot completely map the effective information of high-dimensional data. And the α is the freedom degree of the t-distribution used in the finetuning stage. The larger the value of α, the thinner the tail of the t-distribution. From this, the value of α depends on the size of the crowding problem. A smaller value of α will result in a larger separation between natural clusters, while a larger value of α will cause sample deviations due to abnormal points. Therefore, finding the optimal d and α is particularly important in the parameter t-SNE algorithm.

**Figure 5:**
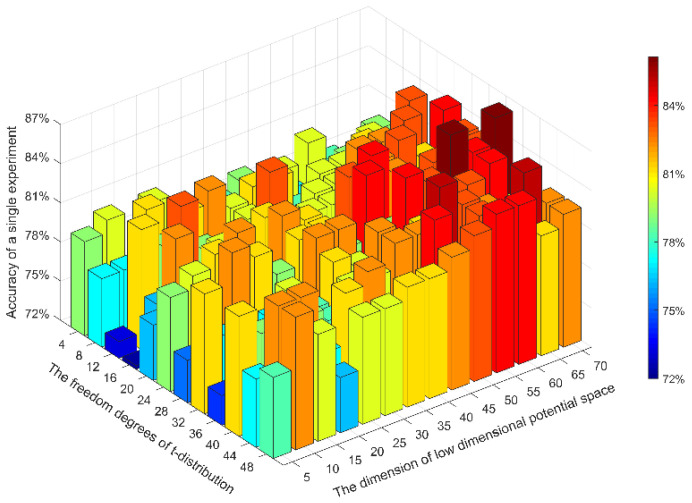
The optimal selection of parameters α and d.

Referring to find the optimal parameter combination, we used the dataset of first subject in BCI competition IV 2a datasets and conducted a traversal search to find the optimal values of d and α. Setting the traversal search range of d to 5–70 with a step size of 5. And the traversal search range of α was 4–48, the step size was 4. Using the proposed deep multi-view feature learning, setting different latent dimensions d and freedom degrees α of t-distribution to calculate the classification accuracy, the results are shown in Figure 5. It can be clearly seen that the greater the dimension d, the higher the accuracy. The best classification result appears when the value of d is 60 or 70. Considering the algorithm efficiency, the latent spatial dimension d = 60 was selected. And the network with the freedom degree α = 32 had the best reduction degree of features. Therefore, the freedom degree of t-distribution was selected as α = 32.

### 2.5. The Selection of Classifer Algorithm

For the deep multi-view features extracted, a suitable classifier algorithm identified their types, as well as SVM was the machine learning algorithm used for classification. Because of its convenient operation and easy processing of small sample datasets, it has been widely used [38,39]. Furthermore, it avoided the course of traditional classification from induction to deduction, realized the efficient transfer from training samples to prediction samples, and simplified the classification process. In addition, its final decision function was only determined by a small number of support vectors, which was conducive to focus our attentions on key samples and eliminate the interference of redundant samples, making the algorithm simpler and more robust. The complexity of its calculation depended on the number of support vector machines instead of the sample space dimension, which avoided the dimensional disaster. Therefore, we chose SVM as a classifier for deep multi-view feature learning in this paper.

## 3. Results

In this section, we resort to the proposed method to test the BCI competition IV 2a datasets. In the experiment, we combined and randomly arranged the training data and the testing data of each subject’s datasets. The 10-fold cross validation method was recommended to obtain accurate evaluation indicators in the experiment. All experiments were conducted with the Matlab 2018b environment, using Intel Core i7–8750h 2.2 GHz with 16 GB of RAM.

The recognition performance of the experimental algorithm was evaluated by the classification accuracy and kappa score [4]. Among them, the classification accuracy rate is the most commonly used metric to evaluate a classifier performance. It was obtained from the confusion matrix by the ratio of the sum of diagonal elements to the total number of samples:

(9) $$A_{cc}=\frac{\sum_{i=1}^{Q}n_{ii}}{N}\times 100\%$$

The kappa score is a useful metric in multi-class problems because it considers not only the correct classification but also the wrong classification. On multi-classification problems, the kappa score reflects the generalization of the model and the consistency in treating different categories [40]. It can be obtained by using the following equation:

(10) $$kappa=\frac{p_{o}-p_{e}}{1-p_{e}}$$

where $p_{o}$ is equal to accuracy, and $p_{e}$ is the sum for the product of the real samples number for each class and the predicted correctly samples number divided by the square of samples number:

(11) $$p_{e}=\frac{a_{1}\times b_{1}+a_{2}\times b_{2}+\cdots +a_{m}\times b_{m}}{n\times n}$$

The actual number of samples in each class is $a_{1}$, $a_{2}$, …, $a_{m}$, respectively, and the number of samples in each class predicted correctly is $b_{1}$, $b_{2}$, …, $b_{m}$, respectively. The class number of samples is m, and n is the total number of samples.

In the experiments of this paper, we extracted the single-view and multi-view features of all subjects in the BCI competition IV 2a dataset, and used SVM to classify these features. The accuracy of classification and kappa score are shown in Table 1. The single-view feature results in the table represent the feature classification results after deep learning when only the time domain, frequency domain or time frequency domain features of the EEG signal were considered. It can be seen from the table that the classification effect when only considering the frequency domain or time-frequency domain characteristics is better than that when only the time domain characteristics were considered on most of the dataset. Only the time domain feature of the dataset provided by the ninth subject is superior to the other two single-view features. This may be due to the difference in the frequency of the EEG signal between this subject and others, resulting in certain frequency characteristics being ignored when extracting its frequency and time-frequency domain features. In addition, we find in the table that the multi-view features classification performance after deep learning is much better than that of single-view features. Especially when the classification effect for single-view features is low, after using the deep multi-view feature learning method, the classification effect is greatly improved. This is due to that the fact single-view features have lost some important relevant information, but the deep multi-view feature learning method can more fully express the relevant motor imagery information.

The recognition accuracy and kappa score of the four-class EEG signals obtained using the deep multi-view feature learning method proposed in this paper are listed in Table 2 and Table 3. In these tables, we compared the method with other methods that deal with the same dataset. It can be seen from the results that using our method to classify motor imagery EEG data had a great performance in accuracy and consistency.

In Table 2, the FBCSP algorithm is one of the earliest methods applied to multi-class motor imagery EEG signals [41]. It is a classic algorithm improved by CSP, which used the SVM classifier for features extracted by FBCSP. SVM-FBCSP is an improved version of FBCSP, which recommended the Hilbert transform to extract the envelope of the signal, and then chose SVM to classify the features [42]. BO is a method to optimize hyperparameters which used Bayesian optimization on the basis of extracting CSP features [43]. Compared with the three algorithms, for the seventh subject, the classification accuracy of the SVM-FBCSP is better than our method, but the average accuracy of our algorithm is 10.7574% higher than FBCSP, 10.3774% higher than BO, and 7.3274% higher than SVM-FBCSP. Aghaei et al. proposed a spatial-spectral generalization common spatial pattern algorithm, called SCSSP [44]. The method considered the spectral characteristics of EEG signals, but our method considered the time domain, frequency domain and time-frequency domain characteristics. On most of the dataset, the classification effect of our method is better than the SCSSP algorithm. but the seventh subject’s classification accuracy is lower than SCSSP, which is only reduced by 3.7162.%. Therefore, the classification accuracy of our method is better than that of other improved CSP algorithms.

Moreover, SS-MEMDBF [45] is a motor imagery task recognition method based on multivariate empirical mode decomposition proposed by Gaur et al. The kappa score of this algorithm is better than our algorithm on the first and fourth datasets, but the kappa score of the algorithm on the second dataset is only 0.24, while the kappa score of our method is 0.4838. This shows that the classification result of SS-MEMDBF algorithm on the second dataset may be biased towards certain types of tasks. In the average of kappa score, our algorithm also offers a certain improvement.

In the Monolithic Network method [46], the author used a variant of discriminative filter bank common spatial pattern (DFBCSP) to extract signal features, and then developed a Bayes-optimized CNN network for classification. After extracting the FBCSP features, the CW-CNN algorithm inputs them into the CNN for classification [42]. The DFFN algorithm is a dense feature fusion convolutional neural network using CSP and CNN technology [7]. These three algorithms adopted CNN to learn the spatial characteristics extracted by CSP. Among them, the average kappa score of the CW-CNN algorithm is the highest, but on the sixth dataset, the kappa score of the algorithm is 0.2690, lower than that of our algorithm which is 0.5301. DFFN has achieved good results on the second and third datasets, but the average accuracy of our algorithm is better than it. The Monolithic network algorithm has the most best classification results, but the average classification accuracy and average kappa score of the algorithm are lower than the method proposed in this article. It can be seen that the classification results obtained by considering the multi-view features of EEG signals and performing deep learning are superior to only using a part of features for deep learning.

Overall, the average classification accuracy of the methods used in this study is 78.5074%, and the average accuracy of other methods is between 65.05%–78.41%, which exceeds all comparison algorithms. The kappa score also surpasses most other methods, having the four best kappa scores, which are provided by subjects 2, 6, 8, and 9 respectively. Therefore, the deep multi-view feature learning algorithm proposed in this paper has a good effect on the recognition of EEG signal motor imagery intention.

In addition, we compared SVM with some currently commonly used classifier algorithms, such as decision tree, linear discriminant analysis (LDA), k-nearest neighbor (KNN), naive Bayesian (NB) and the ensemble classifiers (subspace discriminant (SD)), with the results shown in Table 4. For the average accuracy of different classification algorithms, the performance of KNN and decision tree was ordinary, while LDA, NB, SD, and SVM had good results. Their average accuracies were more than 71.74%, especially SVM and SD that gave 78.5074% and 77.46%, respectively. For each dataset, SVM yielded more optimal classification results, where the improvement on both the second and ninth datasets exceeded 3%. It can be seen that SVM was more suitable to deal with our dataset than other classifiers, it can obtain ideal classification results and solved the need for classifiers. In conclusion, SVM is a better classifier for deep multi-view feature learning algorithms.

## 4. Discussion

In the previous section, we classified and recognized the feature of motor imagery tasks of EEG signals using the deep multi-view feature learning method, and compared the recognition effects with other methods. The comparison results show that, on the datasets provided by most subjects, the features extracted using the deep multi-view feature learning method proposed in this paper are superior to other comparison methods in the classification effect, but referring to the comprehensive evaluation of the designed method, we will discuss it from the following aspects.

**Figure 6:**
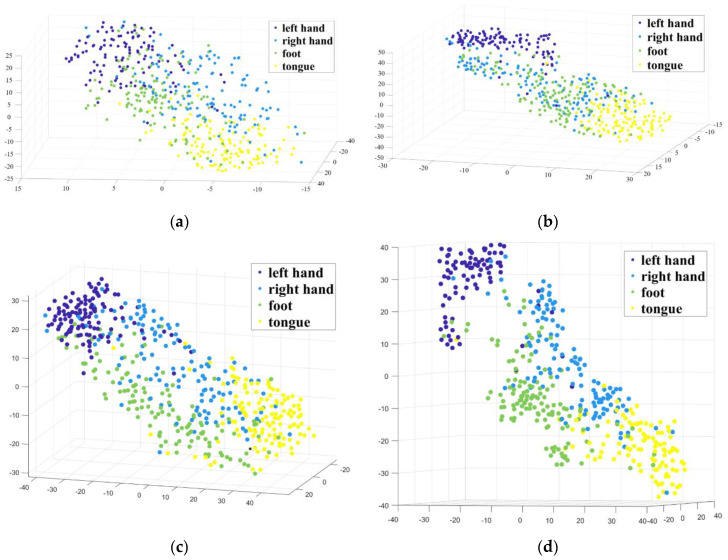
Visualization of single-view and multi-view features of EEG signals: (**a**) Single-view features of time domain signals; (**b**) Single-view features of frequency domain signals; (**c**) Single-view features of time-frequency domain signals; (**d**) Multi-view features.

First, we considered that the loss of information related to motor imagery in EEG signals may lead to a reduction in the classification effect. In order to express the relevant information more completely, we extracted the multi-view features of EEG signals. In Section 3, we compared the classification effects of multi-view and single-view features. Here, we used the t-distributed stochastic neighbor embedding (t-SNE) method to visually analyze the multi-view features F and single-view features, ${F_{1}}^{\prime }$, ${F_{2}}^{\prime }$, and ${F_{3}}^{\prime }$, of the first subject, as shown in Figure 6. The t-SNE is a feature dimensionality reduction visualization algorithm, which maps high-dimensional data to the low-dimensional space through the probability density between them, so the values of the scattered points in the figure do not have numerical significance. The four graphs in Figure 6 are the visualization effects of the four features. It can be seen from the figure that for the same dataset, the distribution positions of the four types of motor imagery tasks are generally consistent in each visualization. In Figure 6a, the distribution of the feature points is scattered, and there are many different types of feature points are overlap together. In Figure 6b, the feature points from four types are closely distributed in a spiral shape. In Figure 6c, the feature points of each class are obviously distributed in different areas. The feature points distribution of the same type is dense, but there is no large distance from other natural clusters. However, in Figure 6d, feature points of each type are distributed closely, and there is a large distance from other types of feature points, and only a few feature points enter into other feature point areas. It can be seen that compared with single-view features, the multi-view feature points of different types are effectively separated, and have better recognizability.

Then, we adopted deep learning algorithms to reduce the dimensions of multi-view features and improve the computational efficiency. This paper used the parameter t-SNE algorithm for deep learning. In the learning process, the algorithm employed the probability distribution between data points to model in the latent space, retained the effective characteristics of the data, removed the influence of redundant features on the network, established an effective separation between natural clusters, solved the crowding problem of potential space, improved the classification accuracy. Its nonlinear characteristics made it not repulsive when embedding high-dimensional nonlinear data in low-dimensional space. In the optimization of the network parameter α, the optimal dimension of the data in the latent space be changed. This may be the reason that the value of the freedom degree α is related to the intrinsic dimension and the latent dimension. When the intrinsic dimension is unchanged, the larger the latent dimension is set, the smaller the optimal freedom degree is. The freedom degree is related to the number of abnormal points. In classification effect, the proposed method was better than some improved CSP feature extraction algorithms and deep learning methods using CNN. This proves that our algorithm had learned the important information about EEG signal motor imagery, which provided a new development direction for EEG signal intention recognition.

Finally, there are still some problems to be solved in the method proposed in this paper. First, in the algorithm, multi-visual feature extraction and the deep learning network are two independent modules. Second, network training requires a large amount of data. When the dataset is too small, it will lead to overfitting, but collecting large amounts of EEG data from each subject is time-consuming. We hope that in some way, when we already have a large amount of data from other subjects, only a small amount of data of current subjects will need to be collected to build an ideal network. Third, our network model is only applicable to the BCI competition IV 2a dataset. When classifying other datasets, the best classification effect may not be obtained because the important parameters in the network have a greater relationship with the intrinsic dimensions and perplexity of the dataset used. We expect these problems to be solved in the future, so deep multi-view feature learning algorithms can be more applied widely.

## 5. Conclusions

In this paper, a feature learning method based on deep multi-view was proposed, and this method was applied to the recognition of motor imagery tasks of EEG signals. According to the characteristics of EEG signals, multi-view features were constructed, and deep feed-forward neural networks were used to learn multi-view features. Using SVM to classify the deep multi-view features obtained by learning, the classification results were compared with several advanced recognition algorithms. The experimental results show that, compared with other algorithms, the recognition accuracy of our method was higher. The visual analysis of deep multi-view features was carried out. The analysis shown that deep multi-view feature learning aggregated the same type of features and discriminated different types of features. It can be seen that the deep multi-view feature learning algorithm proposed in this paper had a great significance in EEG signal motor imagery intention recognition.

## Figures and Tables

**Table 1:** Classification accuracy and kappa score comparison between Multi-view feature and Single-view applied on BCI competition IV dataset 2A.

Methods	Single-View Time Domain	Single-View Frequency Domain	Single-View Time-Frequency	Multi-View
Subject 1	75.8929 (0.6786)	80.3571 (0.7381)	79.4643 (0.7262)	86.6071 (0.8214)
Subject 2	50.4505 (0.3399)	54.9550 (0.3994)	52.2523 (0.3635)	61.2613 (0.4838)
Subject 3	76.3636 (0.6849)	80.9091 (0.7454)	79.0909 (0.7211)	87.2727 (0.7696)
Subject 4	58.8000 (0.4506)	70.8000 (0.6102)	64.6000 (0.5278)	75.2000 (0.6664)
Subject 5	47.2727 (0.2954)	45.4545 (0.2709)	50.9091 (0.3439)	64.5455 (0.5024)
Subject 6	44.3182 (0.2571)	48.8636 (0.3179)	47.7273 (0.3016)	65.9091 (0.5301)
Subject 7	77.4775 (0.6995)	80.1802 (0.7357)	76.5766 (0.6875)	83.7838 (0.7837)
Subject 8	79.8165 (0.7308)	80.7339 (0.7431)	75.2294 (0.6697)	89.9083 (0.8655)
Subject 9	83.1683 (0.7752)	82.1782 (0.7620)	72.2772 (0.6293)	92.0792 (0.8942)
Average	65.9511 (0.5458)	69.3812 (0.5914)	66.4586 (0.5523)	78.5074 (0.6278)

**Table 2:** Classification accuracy comparison with other published results applied on BCI competition IV dataset 2A. The best result for each subject is displayed in bold characters.

Methods	FBCSP [41]	BO [43]	Monolithic Network [46]	FBCSP-SVM [42]	CW-CNN [42]	SCSSP [44]	DFFN [7]	Proposed Methods
Subject 1	76.00	82.12	83.13	82.29	86.11	67.88	83.20	**86.6071**
Subject 2	56.50	44.86	65.45	60.42	60.76	42.18	**65.69**	61.2613
Subject 3	81.25	86.6	80.29	82.99	86.81	77.87	**90.29**	87.2727
Subject 4	61.00	66.28	**81.60**	72.57	67.36	51.77	69.42	75.2000
Subject 5	55.00	48.72	**76.70**	60.07	62.50	50.17	61.65	64.5455
Subject 6	45.25	53.3	**71.12**	44.10	45.14	45.97	60.74	65.9091
Subject 7	82.75	72.64	84.00	86.11	**90.63**	87.5	85.18	83.7838
Subject 8	81.25	82.33	82.66	77.08	81.25	85.79	84.21	**89.9083**
Subject 9	70.75	76.35	80.74	75.00	77.08	76.31	85.48	**92.0792**
Average	67.75	68.13	78.41	71.18	73.07	65.05	76.44	**78.5074**

**Table 3:** Kappa value comparison with other published results applied on BCI competition IV dataset 2A. The best result for each subject is displayed in bold characters.

Methods	SS-MEMDBF [45]	Miao et al. [2]	Monolithic Network [46]	FBCSP-SVM [42]	CW-CNN [42]	sMLR [47]	TSSM-SVM [48]	Proposed Methods
Subject 1	**0.86**	0.6481	0.67	0.7640	0.8150	0.7407	0.70	0.8214
Subject 2	0.24	0.3657	0.35	0.4720	0.4770	0.2685	0.32	**0.4838**
Subject 3	0.70	0.6632	0.65	0.7730	**0.8240**	0.7685	0.75	0.7696
Subject 4	**0.68**	0.5046	0.62	0.6340	0.5650	0.4259	0.54	0.6664
Subject 5	0.36	0.3241	**0.58**	0.4680	0.5000	0.2870	0.32	0.5024
Subject 6	0.34	0.2963	0.45	0.2550	0.2690	0.2685	0.34	**0.5301**
Subject 7	0.66	0.7188	0.69	0.8150	**0.8750**	0.7315	0.70	0.7837
Subject 8	0.75	0.6354	0.70	0.6940	0.7500	0.7685	0.69	**0.8655**
Subject 9	0.82	0.6458	0.64	0.6670	0.6940	0.7963	0.77	**0.8942**
Average	0.60	0.5336	0.59	0.6160	**0.6410**	0.5617	0.571	0.6278

**Table 4:** Accuracy comparison with other classifiers. The best result for each subject is displayed in bold characters.

Methods	Decision Tree	LDA	KNN	NB	SD	SVM
Subject 1	74.5	85.4	75.3	82.7	86.3	**86.6071**
Subject 2	43.9	59.0	47.7	50.5	58.2	**61.2613**
Subject 3	78.8	86.9	77.5	85.6	**89.1**	87.2727
Subject 4	48.8	68.2	50.6	63.9	72.4	**75.2000**
Subject 5	52.2	59.6	55.4	57.4	**65.4**	64.5455
Subject 6	44.2	65.2	53.7	62.7	65.7	**65.9091**
Subject 7	73.2	80.3	72.4	81.2	82.1	**83.7838**
Subject 8	70.8	87.7	75.3	84.1	89.5	**89.9083**
Subject 9	75.6	81.4	77.4	77.6	88.4	**92.0792**
Average	54.64	74.86	65.03	71.74	77.46	78.5074
